# Noninfectious Ascites Neutrophilia in a Patient With Polycythemia Vera

**DOI:** 10.14309/crj.0000000000001364

**Published:** 2024-05-23

**Authors:** Ritika Mazumder, Arjun Chatterjee, William Carey, Shreya Sengupta

**Affiliations:** 1 Department of Internal Medicine, Cleveland Clinic, Cleveland, OH; 2 Department of Gastroenterology and Hepatology, Digestive Disease Institute, Cleveland Clinic, Cleveland, OH

**Keywords:** spontaneous bacterial peritonitis, polycythemia vera, noninfectious ascites, extramedullary hematopoiesis, cirrhosis

## Abstract

A 59-year-old woman with polycythemia vera-related portal hypertension requiring frequent paracentesis was admitted for asymptomatic recurrent spontaneous bacterial peritonitis, which was diagnosed based on elevated polymorphonuclear (PMN) count. She had multiple similar admissions during which she was treated with antibiotics. The patient had chronic baseline leukocytosis due to polycythemia vera. Repeat paracentesis after intravenous antibiotics demonstrated persistent elevation of PMN count without clinical symptoms. A multidisciplinary team concluded that the increased PMN count was secondary to polycythemia. The patient was diagnosed with omental extramedullary hematopoiesis, a rare condition causing elevated PMN count in the absence of bacterial contamination.

## INTRODUCTION

Spontaneous bacterial peritonitis (SBP) is characterized by bacteria in the peritoneal fluid without an apparent source. Ascites fluid cultures often show negative results; therefore, cell analysis is the guideline-recommended method for diagnosing SBP.^1^

Extramedullary hematopoiesis (EH) is a rare condition in which hematopoietic cells, which are normally found in the bone marrow, develop in other organs.^2^ While EH is commonly found in the spleen, liver, and lymph nodes, exceptionally rare sites such as the gastrointestinal tract, prostate, skin, and omentum have also been documented.^3–5^

## CASE REPORT

A 59-year-old woman was admitted with asymptomatic recurrent SBP after large-volume paracentesis. She had a history of JAK2V617F gene mutation and polycythemia vera (PV) leading to deep vein thromboses of the splenic, superior mesenteric, and portal veins. The thromboses were further complicated by noncirrhotic, presinusoidal portal hypertension with bleeding esophageal varices and recurrent ascites, requiring paracentesis every other week. Other medical history included atrial fibrillation, hyperlipidemia, and chronic proximal right-sided celiac artery high-grade stenosis with collateral supply on abdominal imaging.

The patient had multiple consecutive admissions for SBP during which her ascites fluid showed serum-ascites albumin gradient >1.1 g/dL, total ascitic fluid protein of 1.6 g/dL, and a corrected polymorphonuclear (PMN) count ranging from 387 to 659 cells/mm^3^ (ref range: <250 cells/mm^3^, Table 1). The cardiac evaluation involved an echocardiogram, revealing a left ventricular ejection fraction of 66%, with no other significant findings. She had chronic baseline leukocytosis of 15–20 k/μL (ref range: 3.70–11.0 k/μL, attributed to PV), but she remained afebrile, hemodynamically stable, and blood and ascites fluid cultures showed no growth throughout all the admissions. A manual review of the smear revealed rare immature granulocytes without evidence of malignant cells on cytology. Most recently, she presented to the hospital for ascites and underwent large volume paracentesis with laboratory data significant for a corrected PMN count of 387 cells/mm^3^. She was treated with intravenous ceftriaxone daily for 5 days and discharged with once daily ciprofloxacin for secondary prophylaxis. She was readmitted 10 days later after fluid analysis from a routine outpatient paracentesis showed a persistent elevation of PMN count of 374 cells/mm^3^. The patient was asymptomatic, but her ascites fluid laboratory results were concerning for unresolved SBP. The infectious disease team recommended against treating with antibiotics due to low suspicion for ongoing infection and hypothesized that the patient may have a falsely elevated PMN count in the setting of PV. The hematology team evaluated the patient and suspected that omental EH, although rare, was the most likely explanation for this patient's persistently elevated PMN count. Computed tomography scan of her abdomen and pelvis revealed no lesions or pseudo-tumors, rendering it nondiagnostic. Therefore, a clinical diagnosis of EH was made in the setting of her underlying myeloproliferative disorder. The omental EH was postulated to cause elevated leukocytes in the ascites fluid in the absence of bacterial contamination. The patient did not receive any further workup and was monitored clinically without antibiotics. She remained asymptomatic, afebrile, and hemodynamically stable with negative blood and ascites fluid cultures. Moving forward, PMN counts >250 cells/mm^3^ were not treated with antibiotics if the patient was asymptomatic. Following this approach, the patient has not needed any additional hospital admissions at our institution up to now.

**Table 1.** Ascites fluid laboratory results

Ascitic fluid characteristics	June 22, 2022	June 20, 2022	June 10, 2022
Color (nl: yellow)	Bloody	Bloody	Amber
Clarity (nl: clear)	Cloudy	Cloudy	Cloudy
RBC (nl: <2,000/μL)	57,000/μL	68,000/μL	63,000/μL
Total nucleated cells (nl: <1,000/μL)	1,478/μL	1,292/μL	1,047/μL
Neutrophil (nl: 0–1%)	60%	50%	61%
Absolute neutrophil count (PMN)	887/μL	646/μL	639/μL
Corrected neutrophil count^*^ (nl: <250/μL)	659/μL	374/μL	387/μL

* One PMN is subtracted from the absolute PMN count for every 250 red cells/μL.

nl, normal values or range; PMN, polymorphonuclear; RBC, red blood cell.

## DISCUSSION

SBP is a serious complication in individuals with ascites, characterized by infection of ascites fluid without an evident intra-abdominal source. While cirrhosis is a major risk factor for SBP, the condition can also occur with other conditions that lead to ascites, such as heart failure, nephrotic syndrome, Budd-Chiari syndrome, and extrahepatic portal venous obstruction.^6–9^ Patients often present with fever or hypothermia, chills, and abdominal pain, but approximately 30% of patients with SBP are asymptomatic, defined as lack of abdominal pain, fever, or rise in white blood cell (WBC) count above baseline. Ascites fluid analysis, with a diagnostic threshold of PMN count >250, is sufficient for diagnosing SBP since ascitic fluid cultures are frequently nondiagnostic. However, it is essential to consider other conditions that may elevate the PMN count, such as noninfectious inflammatory processes.^1^

We present a case of noninfectious ascites neutrophilia in the setting of presumed omental EH due to PV. EH involves the production of blood cells outside the bone marrow, characterized by ectopic erythropoiesis in the liver or spleen during hypoxia due to heightened erythropoietin production. EH can also occur due to insufficient hematopoiesis in the marrow.

In chronic myeloproliferative disorders such as PV, hematopoietic stem and progenitor cells undergo displacement from the marrow to seek alternative sites for hematopoiesis. In addition, the presence of JAK2V617F mutation, as seen in our patient, is believed to heighten the sensitivity of these cells to growth factors and induce their mobilization to alternative sites.^2,10^ EH usually occurs in the reticuloendothelial system but rarely in areas such as the gastrointestinal tract, central nervous system (CNS), prostate, and skin.^3,5^ This is the fourth reported omental EH case and the first describing associated noninfectious ascites.^3,4,11,12^

EH diagnosis combines clinical assessment, imaging, and sometimes, histopathological examination. Cross-sectional imaging can help visualize and locate extramedullary hematopoietic masses in various organs, and biopsies may be performed to rule out malignancy. No standard EH treatment exists, but managing the primary disease is crucial.^3,5^ Although the computed tomography scan of our patient's abdomen and pelvis did not provide a conclusive diagnosis for EH, her compelling presentation and history of PV led to a clinical diagnosis of omental EH. This unusual condition was responsible for elevated PMNs in the ascites fluid in the absence of bacterial infection.

Noninfectious ascites neutrophilia is an important consideration in patients who are repeatedly hospitalized for asymptomatic SBP. Early identification can prevent multiple courses of unnecessary broad-spectrum antibiotics which put patients at increased risk of adverse effects.^13^ Moving forward for our patient, PMN counts >250 were not treated with antibiotics if the patient was asymptomatic. We propose that in patients with recurrent admissions for asymptomatic SBP, careful correlation of elevated PMNs with clinical symptoms is used to prevent future hospitalizations and unwarranted antibiotic use.

## DISCLOSURES

Author contributions: R. Mazumder: writing the initial draft of the manuscript. A. Chatterjee: drafting and critical review the manuscript. W. Carey and S. Sengupta: final approval of the manuscript, and is the article guarantor.

Financial disclosure: None to report.

Informed consent was obtained for this case report.
